# Proprotein convertase subtilisin/kexin type 9 (PCSK9) Deficiency is Protective Against Venous Thrombosis in Mice

**DOI:** 10.1038/s41598-017-14307-x

**Published:** 2017-10-30

**Authors:** Hui Wang, Qian Wang, Jintao Wang, Chiao Guo, Kyle Kleiman, He Meng, Jason S. Knight, Daniel T. Eitzman

**Affiliations:** 1 Department of Internal Medicine, Cardiovascular Research Center, University of Michigan, Ann Arbor, Michigan USA; 2 Department of Cardiology, the Fourth Affiliated Hospital of China Medical University, Shenyang, China; 3 Division of Rheumatology, Department of Internal Medicine, University of Michigan, Ann Arbor, Michigan USA

## Abstract

The effect of lipid lowering on the incidence of deep venous thrombosis (DVT) is controversial. The purpose of this study was to determine the effect of proprotein convertase subtilisin/kexin type 9 (PCSK9) deficiency on development of DVT in mice. Pcsk9 deficient (*pcsk9* ^−/−^) and wild-type (WT) littermates underwent partial inferior vena cava (IVC) ligation to induce venous thrombosis. 48 hours following IVC ligation, IVC thrombosis was evident in 60% of WT mice and 25% of *pcsk9* ^−/−^ mice (p < 0.05). Analysis of IVC thrombosis revealed greater thrombus weight, length, myeloid cell recruitment, and more neutrophil extracellular trap formation (NETs) in WT compared to *pcsk9* ^−/−^ mice. Intravital microscopy performed two hours following partial IVC ligation revealed that leukocyte firm attachment was increased in WT mice compared to mice undergoing a sham operation, however leukocyte attachment was reduced in *pcsk9* ^−/−^ mice compared to WT mice. In conclusion, deficiency of PCSK9 is associated with protection from venous thrombosis. This protection is associated with reduced leukocyte recruitment and NET formation at the site of thrombosis.

## Introduction

Lipid lowering with statin drugs reduces the risk of myocardial infarction and stroke^1–3^. This reduction in clinical endpoints is associated with a myriad of clinical and preclinical surrogate endpoints suggesting widespread vascular benefits with statin treatment^4–6^. In addition to benefits related to arterial disease, there may also be benefits of lipid lowering towards venous disease. One reported possible additional benefit of statin use is in the prevention of venous thromboembolism (VTE)^7^. In the JUPITER trial, the rates of VTE were 0.18 and 0.32 events per 100 person-years of follow-up in the rosuvastatin and placebo groups, respectively (hazard ratio with rosuvastatin, 0.57; 95% confidence interval [CI], 0.37 to 0.86; P = 0.007)^7^. This beneficial effect on VTE occurred in the absence of any signs of increased bleeding. A case-control study demonstrated that the effect of lipid lowering towards the reduced risk of VTE was not specific to statins^8^. Thus, although pleiotropic effects of statins could account for reduced VTE risk, it is also possible that lipid lowering per se is responsible for this risk reduction and that the observed effects could be related to the potency of the LDL lowering treatment. Inhibition of proprotein convertase subtilisin/kexin type 9 (PCSK9) has emerged as a strategy to markedly reduce LDL with clinical endpoint trials underway to determine efficacy^9^. Preclinical studies have demonstrated reduction of atherosclerosis using murine models of atherosclerosis and PCSK9 deficiency^10^. The purpose of the current study was to determine the effect of PCSK9 on development of venous thrombosis using a model of genetic PCSK9 deficiency.

## Results

### Effect of PCSK9 deficiency on circulating cholesterol levels

To confirm the regulatory effect on lipids by PCSK9 for these experiments, blood lipids were analyzed from plasma samples taken several days before IVC ligation. As expected based on previous studies^11^, levels of total cholesterol, HDL and LDL were markedly reduced in *pcsk9* ^−/−^ mice compared to WT mice. LDL levels were calculated^12^ as they were below limits of assay detection (Table 1).

**Table 1:** Body weight and lipids of WT and *pcsk9* ^−/−^ mice.

	WT	*Pcsk9* ^−/−^
Body weight (g)	24.6 ± 0.6	25.3 ± 0.8
Total cholesterol (mg/dL)	77.1 ± 6.2	25.4 ± 1.9*
HDL (mg/dL)	41.2 ± 3.6	15.3 ± 1.8*
Triglyceride (mg/dL)	30.4 ± 2.4	28.8 ± 4.9
Calculated LDL (mg/dL)	29.8 ± 3.1	4.4 ± 0.9*

*P < 0.001 compared with WT. N = 10 mice per group.

### Effect of PCSK9 deficiency on leukocyte attachment following IVC partial ligation

**Figure 1:**
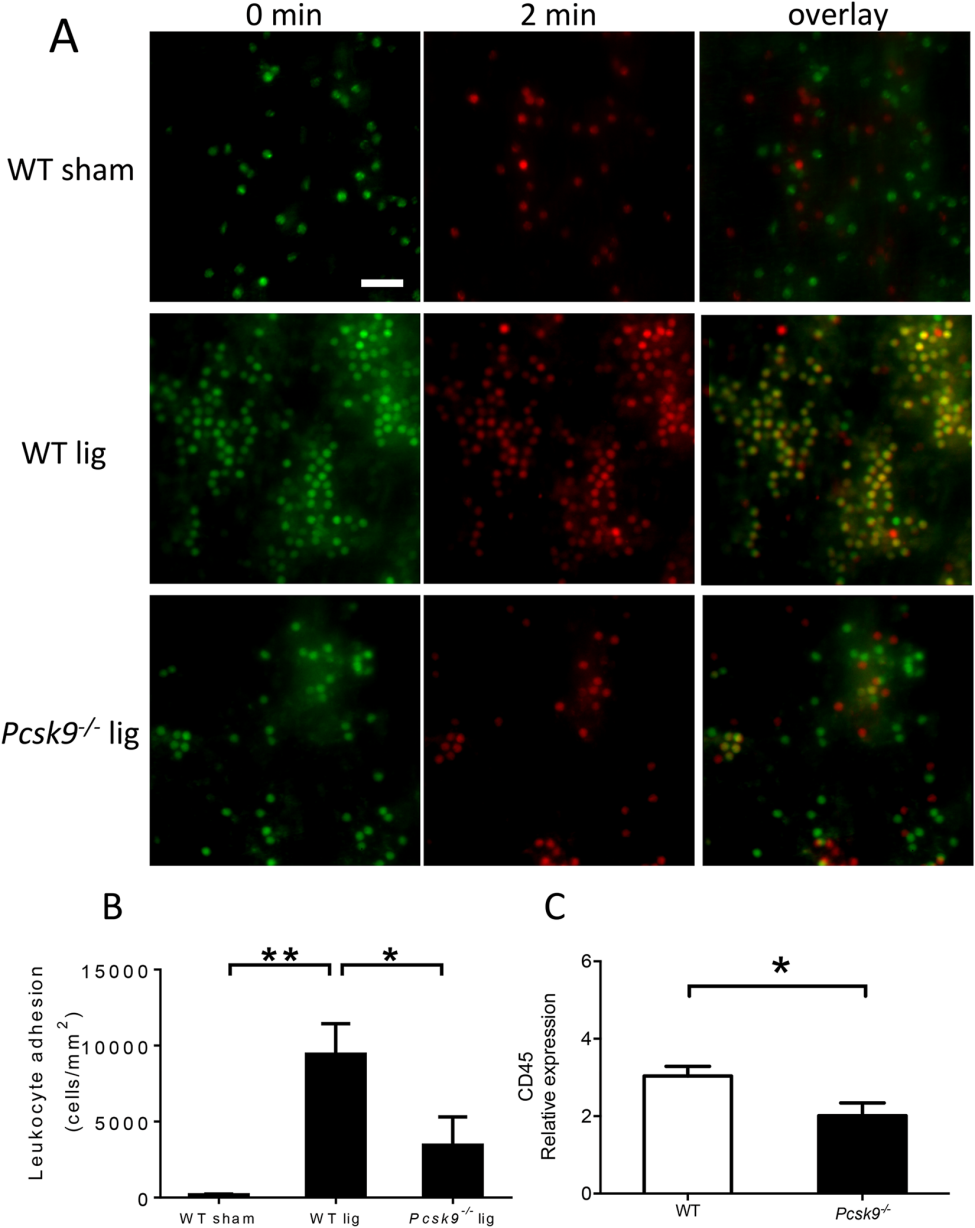
Leukocyte adhesion two hours following IVC ligation (lig) was reduced in *pcsk9* ^−/−^ mice compared to WT mice (n = 4 mice per group). Adherent leukocytes were defined as leukocyte lack of movement for two-minutes and quantified by counting overlaid pictures at 0 min and 2 min. Cells at 0 minutes are green, 2 minutes are red, and cells present at both time points are yellow (**A**) Representative photomicrograph of leukocyte adhesion in sham-operated WT mice (upper panel), WT mice 2 hours following ligation (middle panel), and *pcsk9* ^−/−^ mice 2 hours following ligation (lower panel). (**B**) Quantification of adherent leukocytes expressed as number of attached leukocytes per unit area. *P < 0.05. **P < 0.01. Scale: 50 μm. (**C**) Expression of CD45 in IVC 48 hours after ligation was lower in *pcsk9* ^−/−^ mice compared to WT mice (n = 5 per group). *P < 0.05.

Leukocytes have been shown to be critical mediators of thrombosis in this DVT model. Since LDL may regulate inflammatory responses, mice with low LDL may show a reduced inflammatory response to injuries. To determine whether leukocyte attachment to the IVC was affected in *pcsk9* ^−/−^ mice, intravital microscopy was performed to the site of stasis below the IVC ligation. Two hours following ligation, rhodamine-labeled attached leukocytes were readily apparent although they were reduced in *pcsk9* ^−/−^ mice compared to WT mice (Fig. 1A,B), consistent with a protective, anti-inflammatory effect of PCSK9 deficiency. As a marker of leukocyte accumulation in the vein wall, CD45 expression was determined 48 hours following IVC ligation. CD45 expression was significantly lower in *pcsk9* ^−/−^ mice compared to WT mice (Fig. 1C).

### Effect of PCSK9 deficiency on venous thrombosis following IVC ligation

**Figure 2:**
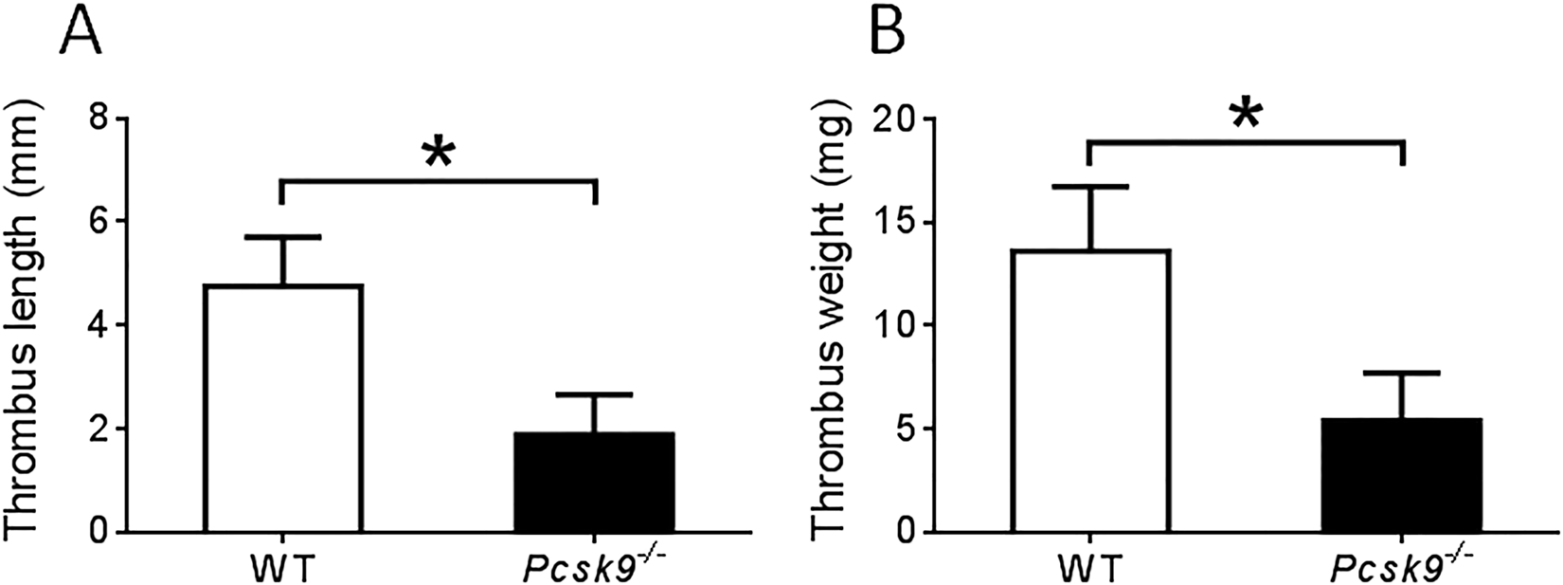
Thrombus formation in inferior vena cava 48 hours after ligation was reduced in *pcsk9* ^−/−^ mice compared to WT mice (n = 20 mice per group). (**A)** Length of the thrombi. B. Weight of the thrombi. *P < 0.05.

**Figure 3:**
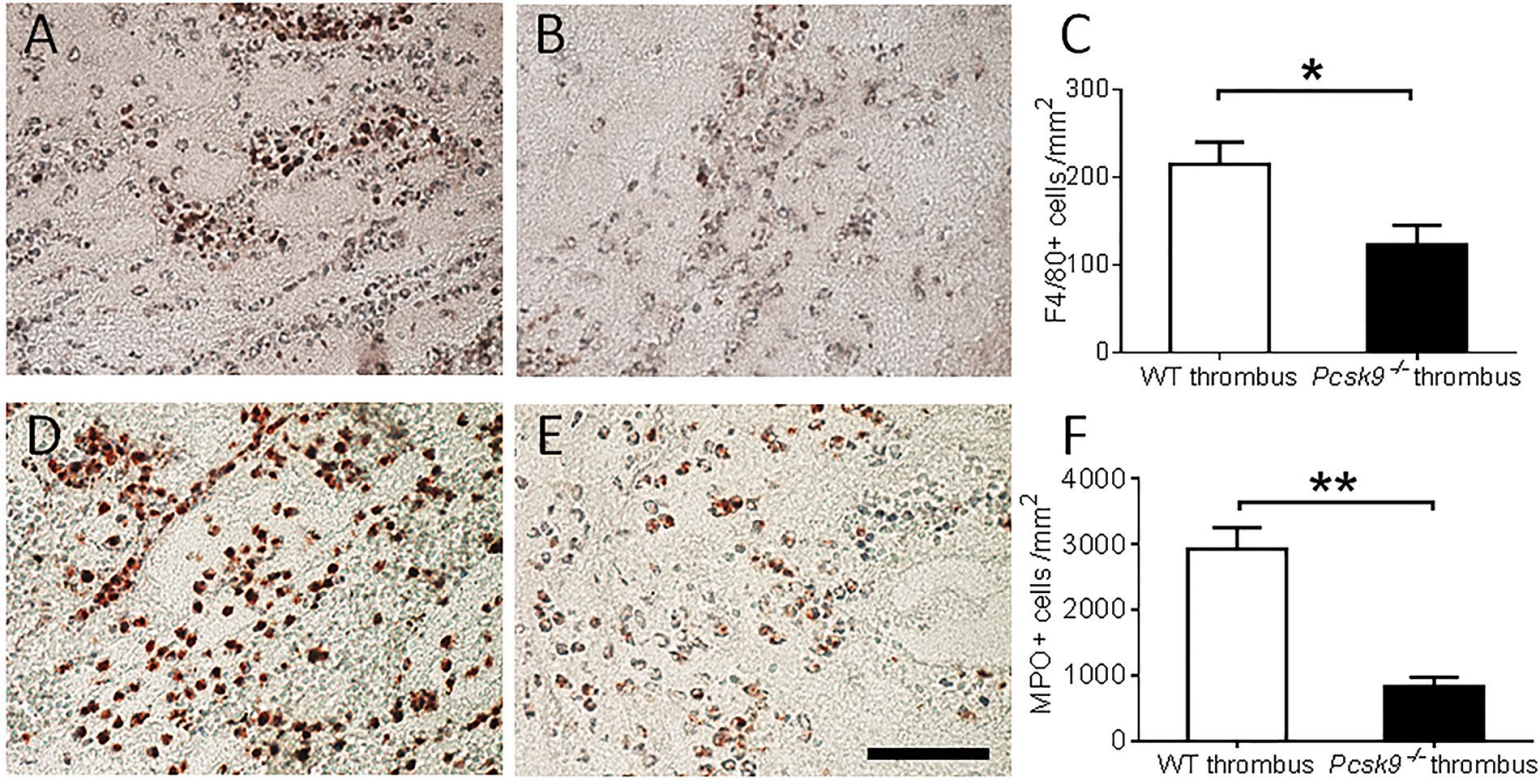
Macrophage and neutrophil accumulation in thrombus 48 hours after IVC ligation were reduced in *pcsk9* ^−/−^ mice compared to WT mice (n = 5 mice per group). (**A**,**B**) Representative photomicrograph of F4/80 staining from WT (**A**) and *pcsk9* ^−/−^ (**B**) mice. (**C**) Quantification of F4/80-positive cells per unit area. (**D**,**E**) Representative photomicrograph of myeloperoxidase (MPO) staining cells from WT (**D**) and *pcsk9* ^−/−^ (**E**) mice. (**F**). Quantification of MPO-positive cells per unit area. *P < 0.05. **P < 0.01. Scale: 50 μm.

**Figure 4:**
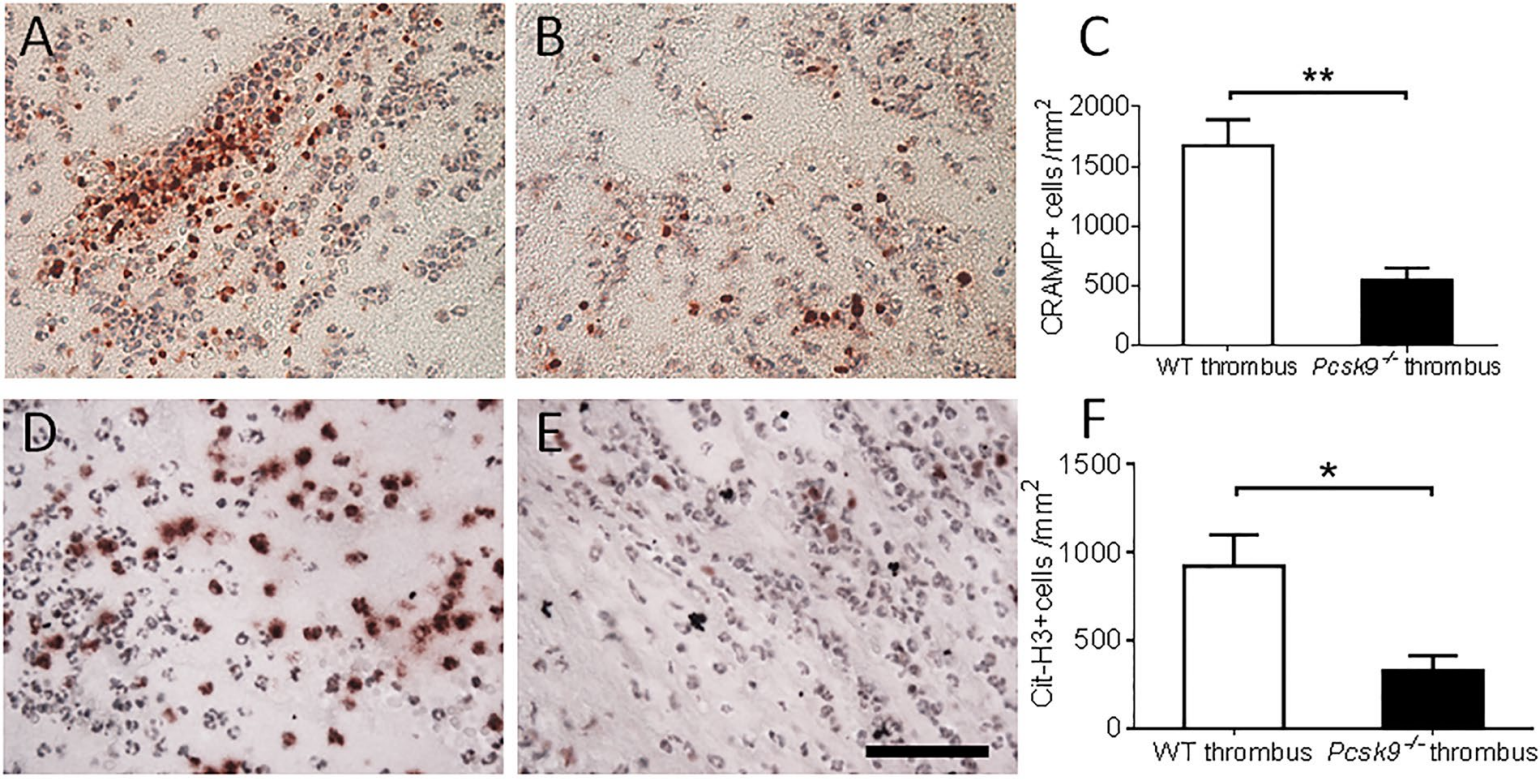
NET formation in thrombi 48 hours after IVC ligation was reduced in *pcsk9* ^−/−^ mice compared to WT mice (n = 5 mice per group). (**A**,**B**) Representative photomicrograph of cathelicidin-related antimicrobial peptide (CRAMP) staining from WT (**A**) and *pcsk9* ^−/−^ (**B**) mice. (**C**) Quantification of CRAMP-positive cells per unit area. (**D**,**E**) Representative photomicrograph of citrullinated histone H3 (Cit-H3) staining from WT (**D**) and *pcsk9* ^−/−^ (**E**) mice. (**F**) Quantification of Cit-H3-positive cells per unit area. *P < 0.05. **P < 0.01. Scale: 50 μm.

48 hours following IVC ligation, visually apparent thrombus was evident in 12 of 20 (60%) WT mice but in only 5 of 20 (25%) *pcsk9* ^−/−^ mice, p < 0.05. Accordingly, the weight and length of IVC thrombi were reduced in *pcsk9* ^−/−^ mice compared to WT mice (Fig. 2). Formalin fixed thrombi were also analyzed for cellular composition. Immunohistochemical staining showed evidence of increased macrophage and especially neutrophil accumulation in the thrombi of WT mice compared to *pcsk9* ^−/−^ mice (Fig. 3). As this DVT model has been shown to exhibit NETosis that contributes to clot formation, immunostaining for CRAMP and Cit-H3 was performed which was greater in WT mice (Fig. 4), suggesting reduced NETosis in *pcsk9* ^−/−^ mice.

**Figure 5:**
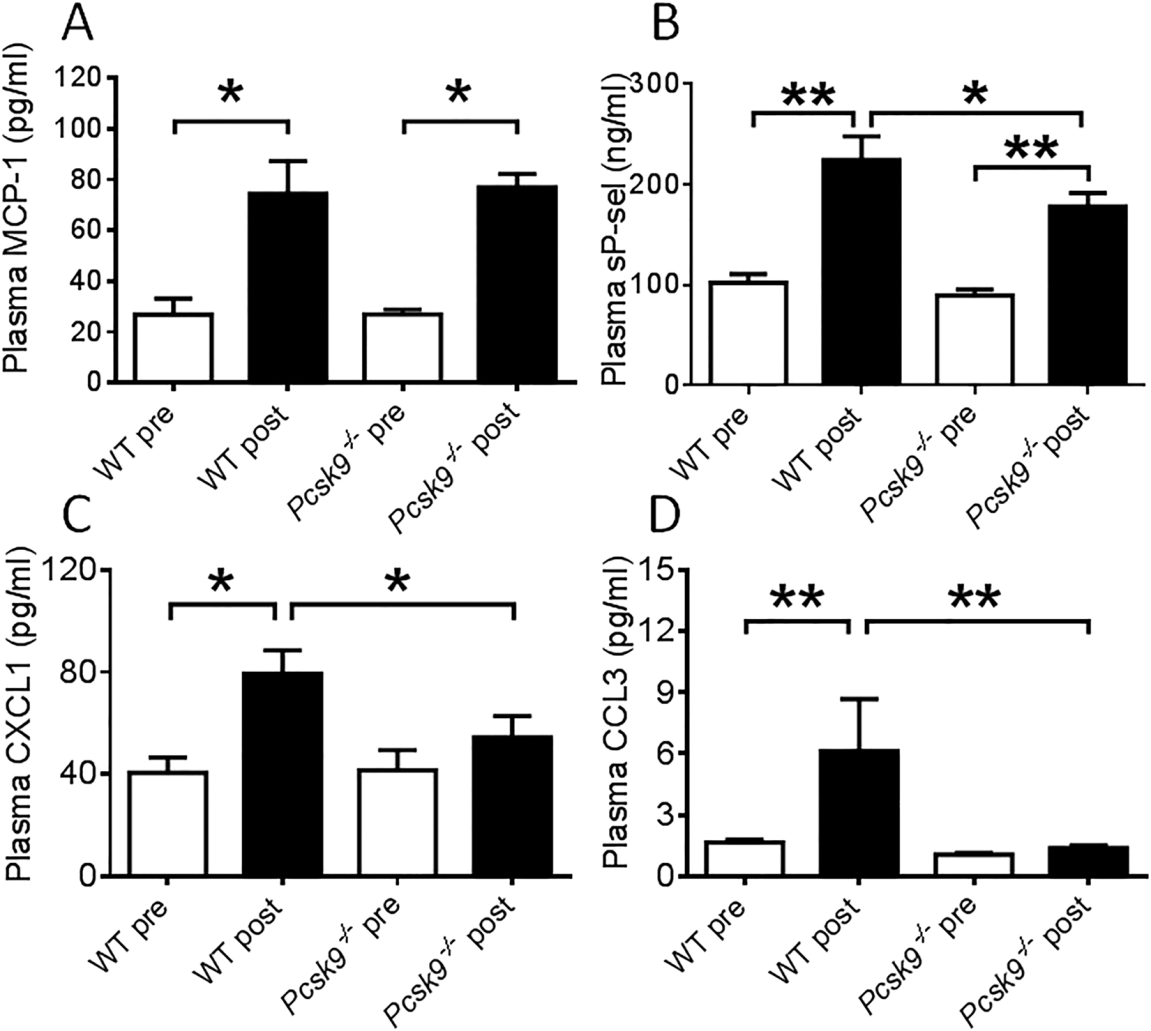
Measurement of plasma factors in WT and *pcsk9* ^−/−^ mice before and 48 hours after IVC ligation (n = 8 mice per group). (**A**) Levels of monocyte chemoattractant protein-1 (MCP-1). (**B**) Levels of soluble P-selectin (sP-sel). (**C**) Levels of chemokine (C-X-C motif) ligand 1 (CXCL1). (**D**) Levels of chemokine (C-C motif) ligand 3 (CCL3). *P < 0.05. **P < 0.01.

Potential circulating mediators of leukocyte chemoattraction and adhesion implicated in inflammatory vascular diseases were measured before and at the termination of the thrombosis experiment. MCP-1 levels were significantly elevated following IVC ligation but were not different between *pcsk9* ^−/−^ mice and WT mice (Fig. 5A). In contrast, levels of sP-sel, CXCL1 and CCL3 were all reduced in *pcsk9* ^−/−^ mice compared to WT mice following IVC ligation (Fig. 5B–D).

Circulating platelet counts were not different between WT (863.3 ± 73.6 × 10^9^/L) and *pcsk9* ^−/−^ mice (981.6 ± 200.7 × 10^9^/L), p > 0.05. Whole blood aggregation area under the curve (AUC) values were also not different between WT and *pcsk9* ^−/−^ mice: 201.1 ± 9.7 AU vs 196.0 ± 14.4 AU, respectively, p > 0.05.

Total white blood cell counts were higher in *pcsk9* ^−/−^ compared to WT mice (WT = 6.16 ± 2.73 vs 12.27 ± 1.89 × 10^9^/L in *pcsk9* ^−/−^ mice, p < 0.0001). This was predominantly due to increased numbers of neutrophils (WT = 1.57 ± 0.89 vs 2.95 ± 1.41 × 10^9^/L in *pcsk9* ^−/−^ mice, p < 0.05) and lymphocytes (WT = 4.19 ± 2.07 vs 8.72 ± 1.70 × 10^9^/L in *pcsk9* ^−/−^ mice, p < 0.0002). Monocytes were not significantly different between the groups (WT = 0.37 ± 0.45 vs 0.55 ± 0.20 × 10^9^/L in *pcsk9* ^−/−^ mice, p > 0.05) nor were erythrocytes (WT = 8.25 ± 0.37 vs 8.61 ± 0.30 × 10^12^/L in *pcsk9* ^−/−^ mice, p > 0.05).

## Discussion

VTE, including DVT and pulmonary embolism (PE), is a common cause of morbidity and mortality^13^. Current treatment strategies involve drugs that inhibit factors in the coagulation cascade^14^, which can lead to bleeding complications. Any drug useful in prevention of VTE, without the concomitant bleeding risk, could be clinically useful. Therefore, identification of risk factors for VTE distinct from the coagulation cascade is a high priority. The association between hyperlipidemia and VTE risk is controversial with some studies showing positive associations^15–17^ and others neutral^18^. In support of a causal relationship between lipid levels and VTE, some trials of lipid lowering with statins have shown significant reductions in the incidence of VTE^7^. Multiple mechanism(s) could contribute to the link between lipids and reduced VTE risk including effects on platelet function^19–21^, changes in coagulation factors^16,22^, alterations in fibrinolysis^23,24^, and effects on inflammatory pathways^25,26^.

PCSK9 is a protease that targets the LDL receptor for degradation^27^. Humans with gain-of-function PCSK9 mutations have hyperlipidemia and heightened cardiovascular risk while those with loss-of-function mutations are protected from complications of atherosclerosis^27–29^. Clinical trials with antibodies have established their efficacy in reducing LDL^9^ and clinical trials are now underway to determine their effects on cardiovascular complications. A recently published IVUS trial strongly supports a beneficial effect of PCSK9 inhibition on progression of atherosclerosis^30^.

Similar to statins, PCSK9 inhibition leads to upregulation of LDL receptors with resulting enhanced LDL clearance^27^. Mice with PCSK9 deficiency appear to have a similar phenotype to PCSK9 deficient humans with low levels of LDL^11^ and protection from atherosclerosis^10^. Since lipid reduction may attenuate chronic inflammatory responses in arterial disease, reduced lipid levels may also have beneficial effects on inflammatory responses in venous disease. To determine whether the vascular benefits of PCSK9 inhibition might extend to venous disease, we tested the effects of PCSK9 deficiency in a mouse model of DVT. Partial ligation of the mouse IVC leads to occlusive thrombosis in the majority of mice on the C57BL6/J strain background at 48 hours following ligation^31^. This relatively chronic model induced by venous stasis may be particularly relevant to most DVT’s that occur in humans. DVT in this model has been shown to involve leukocytes, platelets, coagulation factors and neutrophil extracellular traps^31^.

As expected, mice deficient in PCSK9 in this study showed reductions in total cholesterol, LDL, and HDL compared to control mice, even on standard chow diet. Circulating lipid levels play a critical role in inflammatory responses. For example, in mouse models of atherosclerosis, hyperlipidemia leads to marked upregulation of leukocyte-endothelial interactions that are increased even further in response to tissue injury or hemodynamic perturbations^32–34^. Whether very low cholesterol levels lead to reduced L-E interactions in a non-hyperlipidemic model is less clear. In the current study, partial ligation of the IVC triggered an increase in leukocyte adhesion inferior to the ligation as has been reported by others^31^. Leukocyte adhesion was reduced in mice with PCSK9 deficiency suggesting a beneficial role of very low LDL levels towards leukocyte recruitment in this model. However, we cannot rule out the possibility that other lipid-independent effects of PCSK9 could be affecting the phenotype in this model. Leukocyte accumulation in this model has been previously shown to play an important contributory role toward the subsequent development of stable venous thrombosis^31^. Consistently, mice with PCSK9 deficiency developed less IVC thrombosis compared to wild-type mice at 48 hours. Thrombi from PCSK9 deficient mice were also characterized by reduced myeloid markers. Leukocyte recruitment in this model is p-selectin dependent and associated with enhanced CXCL1 expression^30,31^. CXCL1 expression and release have previously been shown to be modified by lipids^35^. It could be that the altered circulating lipid profile in PCSK9 deficient mice might reduce expression and/or release of this neutrophil chemoattractant. In this study plasma levels of sP-selectin and CXCL1 were reduced in PCSK9 deficient mice. Neutrophils have been shown to promote thrombosis through generation of NETs^36^. Thrombi present in PCSK9 deficient mice showed reduced NET formation. Since NET formation has been shown by several groups to promote venous thrombosis, it is likely that by affecting myeloid cell recruitment following IVC stasis, PCSK9 deficiency leads to reduced subsequent NET formation. It is also possible that other undefined mechanisms play a role in this process. Although we did not observe an effect of PCSK9 deficiency on whole blood platelet aggregation, a recent paper reported an association between elevated PCSK9 levels and platelet reactivity in patients with acute coronary syndromes^37^. Unexpectedly, circulating neutrophil and lymphocyte counts were elevated in PCSK9 deficient mice, an observation that will require further study.

In summary, this study supports the hypothesis that the vascular benefits of lipid lowering therapy may extend to venous disease. Human data should be revealing with regards to arterial and venous endpoints in ongoing clinical trials of PCSK9 inhibition.

## Methods

### Animals

Male PCSK9 deficient (*pcsk9* ^−/−^) mice on the C57BL6/J strain background were purchased from Jackson Laboratory (Bar Harbor, Maine)^11^. Then male *pcsk9* ^−/−^ mice were crossbred to female *pcsk9* ^*+/+*^ C57BL6/J mice (Jackson Laboratory, Bar Harbor, Maine) to generate *pcsk9* ^+/−^ mice. *Pcsk9* ^−/−^ and *pcsk9* ^*+/+*^ (wild-type, WT) mice were generated from *pcsk9* ^+/−^ intercrosses. Male *pcsk9* ^−/−^ and WT littermates from multiple simultaneous breedings were used for experiments. Mice were housed under specific pathogen-free conditions in static microisolator cages with tap water ad libitum in a temperature-controlled room with a 12:12-hour light/dark cycle and were fed a standard laboratory rodent diet (No. 5001, TestDiet, Richmond, IN).

All animal use protocols complied with the Principle of Laboratory and Animal Care established by the National Society for Medical Research and were approved by the University of Michigan Committee on Use and Care of Animals. All animal experiments were performed according to the National Institutes of Health Guide for the Care and Use of Laboratory Animals.

### Model of inferior vena cava (IVC) thrombosis

A stenosis model of deep venous thrombosis (DVT) was performed on male WT and *pcsk9* ^−/−^ mice at 10 weeks of age. Mice were anesthetized with intraperitoneal (i.p.) injection of sodium pentobarbital (67 mg/kg). The surgical procedure was performed as described previously^38,39^. Briefly, the abdomen was opened through a ventral midline laparotomy. The small intestine was gently moved aside and draped with sterile saline-soaked gauze. The IVC was exposed gently and a metal spacer made from 30-gauge needle was placed on the outside of vessel. The IVC and spacer were ligated using a 6.0 nylon suture (Ethicon, Somerville, NJ) just below the left renal vein. The spacer was then removed to allow blood flow through the IVC. Side branches of the IVC were not ligated or manipulated during the procedure. For sham-operated mice, the IVC was exposed but no ligation was performed. 48 hours after surgery, mice were sacrificed with intraperitoneal (i.p.) injection of sodium pentobarbital (100 mg/kg). Presence, weight and length of thrombi from IVC were measured. Mice without thrombus were assigned a value of 0 for analysis of length and weight. Plasma samples for analysis were collected via retro-orbital bleeding before procedures and 48 hours after procedures via cardiac puncture prior to euthanasia.

### IVC intravital microscopy

Leukocyte-endothelial interactions were measured similar to our previously described experiments involving cremasteric venules^40^. For IVC analysis, 200 µl rhodamine-6G (0.067 mg/ml) was injected intravenously via the tail vein 2 hours following partial IVC ligation. The abdomen was then reopened and labeled leukocytes were visualized with a Nikon FN1 fixed-stage microscopy system with X-cite for epi-fluorescence. The area of interest consisted of a 2-3 mm segment of IVC distal to the IVC ligation. Videos were recorded with a Photometrics Coolsnap Cascade 512B color digital camera system for 2 minutes and analyzed with the MetaMorph Premier software package (Molecular Devices). After intravital microscopy, mice were sacrificed with intraperitoneal (i.p.) injection of sodium pentobarbital (100 mg/kg). Adherent cells were defined by lack of movement for at least 2 minutes. The number of adherent cells was manually counted as overlaid cells (yellow) in pictures taken at 0 min (assigned green) and 2 min (assigned red) of recording.

### Real-Time Polymerase Chain Reaction

RNA from the IVC was isolated using a QIAGEN RNeasy Mini Kit (QIAGEN Inc., Valencia, CA). The primer sets were purchased from Applied Biosystems (Carlsbad, CA). RTPCR was performed using an ABI Prism 7000 Sequence Detection System (Applied Biosystems, Carlsbad, CA). 100 ng of RNA and 1 μl of primer were used per reaction. 7000 System SDS Software and the 2^−ΔΔCT^ method^41^ were used to analyze the results. Results were presented as fold change of transcripts for target normalized to internal control (GAPDH).

### Immunohistochemistry

48 hours following IVC ligation, the formed thrombi were isolated and fixed in zinc formation. Cellular components of thrombi were determined using corresponding antibodies on paraffin-embedded sections. Macrophages were identified with a rat anti-mouse F4/80 monoclonal antibody (1:100) (Abcam, Cambridge, MA). Neutrophils were identified with a rabbit anti-mouse myeloperoxidase (MPO) polyclonal antibody (1:200) (DAKO, Carpinteria, CA). The NET-related markers cathelicidin-related antimicrobial peptide (CRAMP) and citrullinated histone H3 (Cit-H3) were detected using a rabbit anti-mouse CRAMP polyclonal antibody (1:200) (Innovagen, Lund, Sweden) and a rabbit anti-mouse Cit-H3 polyclonal antibody (1:100) (Abcam, Cambridge, MA) as described previously^42^. Positive cells were detected with corresponding biotin-conjugated secondary antibodies. Stained cells were counted manually from five positively stained fields in each section using NIH ImageJ software and expressed as a percentage of positive cells per unit area.

### Measurement of plasma factors

To measure cholesterol and triglycerides levels, plasma samples were collected via retro-orbital bleeding using capillary tubes from WT and *pcsk9* ^−/−^ mice at 10 weeks of age before procedures. The samples were analyzed in the Chemistry Core of the Michigan Diabetes Research and Training Center using Enzymatic-Colorimetric kits (Roche, Indianapolis, IN).

Commercial enzyme-linked immunosorbent assay (ELISA) kits (R&D Systems, Minneapolis, MN) were used to measure circulating concentrations of monocyte chemoattractant protein-1 (MCP-1), soluble P-selectin (sP-sel), chemokine (C-X-C motif) ligand 1 (CXCL1), and Chemokine (C-C motif) ligand 3 (CCL3) in plasma samples obtained before and after procedures as previously described^43^.

### Cell counts and Whole blood aggregation

Complete circulating blood cell and platelet counts were obtained using a Hemavet 950 haematology system (Drew Scientific, Miami Lakes, Florida). For whole blood aggregation, 500μl of whole blood anticoagulated with 3.2% sodium citrate (1: 9) was diluted in same volume of 0.9% saline and incubated for 5 min at 37 °C. Collagen (5μg/ml) - stimulated whole blood aggregation was performed using a Whole Blood / Optical Lumi- Aggregation System (Chrono-Log Corp., Havertown, PA) according to manufacturer’s instruction.

### Statistical analysis

All data are presented as mean ± standard error. Statistical analysis was carried out using GraphPad Prism. To compare incidence of DVT in WT and *pcsk9* ^−/−^ mice, the chi-square test was performed. For comparison between two groups, results were analyzed using unpaired t-test. For multiple comparisons, results were analyzed using one-way ANOVA followed by Tukey post-test analysis. Probability values of p < 0.05 were considered statistically significant.

## References

[1] LaRosa JC (2005). Intensive lipid lowering with atorvastatin in patients with stable coronary disease. N Engl J Med.

[2] Trialists CT, C. (2010). Efficacy and safety of more intensive lowering of LDL cholesterol: a meta-analysis of data from 170,000 participants in 26 randomised trials. Lancet.

[3] Ridker PM (2008). Rosuvastatin to prevent vascular events in men and women with elevated C-reactive protein. N Engl J Med.

[4] Davignon J (2004). Beneficial cardiovascular pleiotropic effects of statins. Circulation.

[5] Schonbeck U, Libby P (2004). Inflammation, immunity, and HMG-CoA reductase inhibitors: statins as antiinflammatory agents?. Circulation.

[6] Stancu C, Sima A (2001). Statins: mechanism of action and effects. J Cell Mol Med.

[7] Glynn RJ (2009). A randomized trial of rosuvastatin in the prevention of venous thromboembolism. N Engl J Med.

[8] Ashrani AA (2015). Is lipid lowering therapy an independent risk factor for venous thromboembolism? A population-based case-control study. Thromb Res.

[9] Blom DJ (2014). A 52-week placebo-controlled trial of evolocumab in hyperlipidemia. N Engl J Med.

[10] Denis M (2012). Gene inactivation of proprotein convertase subtilisin/kexin type 9 reduces atherosclerosis in mice. Circulation.

[11] Rashid S (2005). Decreased plasma cholesterol and hypersensitivity to statins in mice lacking Pcsk9. Proc Natl Acad Sci USA.

[12] Friedewald WT, Levy RI, Fredrickson DS (1972). Estimation of the concentration of low-density lipoprotein cholesterol in plasma, without use of the preparative ultracentrifuge. Clin Chem.

[13] Goldhaber SZ, Bounameaux H (2012). Pulmonary embolism and deep vein thrombosis. Lancet.

[14] Marik PE, Cavallazzi R (2015). Extended Anticoagulant and Aspirin Treatment for the Secondary Prevention of Thromboembolic Disease: A Systematic Review and Meta-Analysis. PLoS One.

[15] Kawasaki T (1997). Hypercholesterolemia as a risk factor for deep-vein thrombosis. Thromb Res.

[16] Griffin JH, Fernandez JA, Deguchi H (2001). Plasma lipoproteins, hemostasis and thrombosis. Thromb Haemost.

[17] Vaya A (2002). Hyperlipidaemia and venous thromboembolism in patients lacking thrombophilic risk factors. Br J Haematol.

[18] Goldhaber SZ (1997). A prospective study of risk factors for pulmonary embolism in women. JAMA.

[19] Pathansali R, Smith N, Bath P (2001). Altered megakaryocyte-platelet haemostatic axis in hypercholesterolaemia. Platelets.

[20] Eitzman DT, Westrick RJ, Xu Z, Tyson J, Ginsburg D (2000). Hyperlipidemia promotes thrombosis after injury to atherosclerotic vessels in apolipoprotein E-deficient mice. Arterioscler Thromb Vasc Biol.

[21] de Man FH (2000). Activated platelets in patients with severe hypertriglyceridemia: effects of triglyceride-lowering therapy. Atherosclerosis.

[22] Bladbjerg EM, Marckmann P, Sandstrom B, Jespersen J (1994). Non-fasting factor VII coagulant activity (FVII:C) increased by high-fat diet. Thromb Haemost.

[23] Sartori MT (2003). The PAI-1gene 4G/5G polymorphism and deep vein thrombosis in patients with inherited thrombophilia. Clinical and applied thrombosis/hemostasis: official journal of the International Academy of Clinical and Applied Thrombosis/Hemostasis.

[24] Han P (1988). Altered fibrinolysis in DVT: influence of site of sampling. Thromb Haemost.

[25] Krauzova E (2016). Acute hyperlipidemia initiates proinflammatory and proatherogenic changes in circulation and adipose tissue in obese women. Atherosclerosis.

[26] Dhanesha N (2015). Genetic Ablation of Extra Domain A of Fibronectin in Hypercholesterolemic Mice Improves Stroke Outcome by Reducing Thrombo-Inflammation. Circulation.

[27] Abifadel M (2014). Living the PCSK9 adventure: from the identification of a new gene in familial hypercholesterolemia towards a potential new class of anticholesterol drugs. Curr Atheroscler Rep.

[28] Cohen J (2005). Low LDL cholesterol in individuals of African descent resulting from frequent nonsense mutations in PCSK9. Nature genetics.

[29] Cohen JC, Boerwinkle E, Mosley TH, Hobbs HH (2006). Sequence variations in PCSK9, low LDL, and protection against coronary heart disease. N Engl J Med.

[30] Nicholls, S.J. *et al*. Effect of Evolocumab on Progression of Coronary Disease in Statin-Treated Patients: The GLAGOV Randomized Clinical Trial. *JAMA* (2016). 10.1001/jama.2016.16951 27846344

[31] von Bruhl ML (2012). Monocytes, neutrophils, and platelets cooperate to initiate and propagate venous thrombosis in mice *in vivo*. J Exp Med.

[32] Combadiere C (2008). Combined inhibition of CCL2, CX3CR1, and CCR5 abrogates Ly6C(hi) and Ly6C(lo) monocytosis and almost abolishes atherosclerosis in hypercholesterolemic mice. Circulation.

[33] Wright AP (2010). Atherosclerosis and leukocyte-endothelial adhesive interactions are increased following acute myocardial infarction in apolipoprotein E deficient mice. Atherosclerosis.

[34] Dutta P (2012). Myocardial infarction accelerates atherosclerosis. Nature.

[35] Chan KL (2015). Palmitoleate Reverses High Fat-induced Proinflammatory Macrophage Polarization via AMP-activated Protein Kinase (AMPK). J Biol Chem.

[36] Kimball AS, Obi AT, Diaz JA, Henke PK (2016). The Emerging Role of NETs in Venous Thrombosis and Immunothrombosis. Front Immunol.

[37] Navarese EP (2017). Association of PCSK9 with platelet reactivity in patients with acute coronary syndrome treated with prasugrel or ticagrelor: The PCSK9-REACT study. Int J Cardiol.

[38] Brill A (2011). von Willebrand factor-mediated platelet adhesion is critical for deep vein thrombosis in mouse models. Blood.

[39] Meng, H. *et al*. *In vivo* role of neutrophil extracellular traps in antiphospholipid antibody-mediated venous thrombosis. *Arthritis & rheumatology* (2016). 10.1002/art.39938 PMC5329054 27696751

[40] Luo W (2012). P-selectin glycoprotein ligand-1 inhibition blocks increased leukocyte-endothelial interactions associated with sickle cell disease in mice. Blood.

[41] Livak KJ, Schmittgen TD (2001). Analysis of relative gene expression data using real-time quantitative PCR and the 2(-Delta Delta C(T)) Method. Methods.

[42] Brinkmann V, Abu Abed U, Goosmann C, Zychlinsky A (2016). Immunodetection of NETs in Paraffin-Embedded Tissue. Front Immunol.

[43] Russo HM (2010). P-selectin glycoprotein ligand-1 regulates adhesive properties of the endothelium and leukocyte trafficking into adipose tissue. Circ Res.

